# Alternative splicing tends to avoid partial removals of protein-protein interaction sites

**DOI:** 10.1186/1471-2164-14-379

**Published:** 2013-06-07

**Authors:** Alessio Colantoni, Valerio Bianchi, Pier Federico Gherardini, Gianpaolo Scalia Tomba, Gabriele Ausiello, Manuela Helmer-Citterich, Fabrizio Ferrè

**Affiliations:** 1 Centre for Molecular Bioinformatics, Department of Biology, University of Rome Tor Vergata, Via della Ricerca Scientifica snc, 00133 Rome, Italy; 2 Department of Mathematics, University of Rome Tor Vergata, Via della Ricerca Scientifica snc, 00133 Rome, Italy; 3 Current address: Center for Genomic Science of IIT@SEMM, Istituto Italiano di Tecnologia, via Adamello 16, 20139 Milan, Italy; 4 Current address: Department of Microbiology & Immunology, Baxter Laboratory for Stem Cell Biology, Stanford University School of Medicine, Stanford, California, USA

**Keywords:** Alternative splicing, Protein-protein interaction, Hot spots, Protein three-dimensional structure, Disordered regions

## Abstract

**Background:**

Anecdotal evidence of the involvement of alternative splicing (AS) in the regulation of protein-protein interactions has been reported by several studies. AS events have been shown to significantly occur in regions where a protein interaction domain or a short linear motif is present. Several AS variants show partial or complete loss of interface residues, suggesting that AS can play a major role in the interaction regulation by selectively targeting the protein binding sites. In the present study we performed a statistical analysis of the alternative splicing of a non-redundant dataset of human protein-protein interfaces known at molecular level to determine the importance of this way of modulation of protein-protein interactions through AS.

**Results:**

Using a Cochran-Mantel-Haenszel chi-square test we demonstrated that the alternative splicing-mediated partial removal of both heterodimeric and homodimeric binding sites occurs at lower frequencies than expected, and this holds true even if we consider only those isoforms whose sequence is less different from that of the canonical protein and which therefore allow to selectively regulate functional regions of the protein. On the other hand, large removals of the binding site are not significantly prevented, possibly because they are associated to drastic structural changes of the protein. The observed protection of the binding sites from AS is not preferentially directed towards putative hot spot interface residues, and is widespread to all protein functional classes.

**Conclusions:**

Our findings indicate that protein-protein binding sites are generally protected from alternative splicing-mediated partial removals. However, some cases in which the binding site is selectively removed exist, and here we discuss one of them.

## Background

Protein function is often modulated by binding with specific partners. Protein-protein interactions can be regulated by post-translational modifications, such as phosphorylation [1-3], or via binding with small ligands [4,5]. Moreover many examples are known of protein-protein interactions that are regulated via AS [6,7]. AS can modulate interactions by: i) removing binding sites or domains; ii) inserting a stretch of sequence that disrupts binding; iii) modulating the inclusion of a specific binding motif. Several studies have attempted to assess how widespread these forms of regulation are. The overall impact of alternative splicing on protein-protein interaction domains was investigated in a work published in 2004 [8], which indicates that the majority of the domains that are removed by alternative splicing at high frequencies are protein-protein interaction domains. The authors analyzed 13384 protein isoforms of 4442 genes and found that 50 protein domain types were significantly removed by alternative splicing at higher frequencies; this list of domain types was enriched in domains involved in protein-protein interactions. The effects of AS on the inclusion or exclusion of short interacting modules have also been studied in a systematic way [9]. The analysis of 1421 protein isoforms produced from 404 genes showed that regions that are alternatively spliced are enriched in short linear motifs that are typically involved in low-affinity interactions [10]. Ellis and coworkers also found that linear motifs are enriched within tissue-specific alternative coding exons [11]. They employed a splicing variant-specific form of the co-immunoprecipitation assay to demonstrate the extensive and tissue-specific rewiring of protein interaction networks mediated by the inclusion of specific alternative exons. Another way, though less commonly observed, through which protein-protein interactions can be modulated by alternative splicing is the selective (complete or partial) removal of the region that physically interacts with the partner—the binding site or interface, which may be part of a domain or not, and which often does not coincide exactly with a linear motif [12-16].

In this work we investigated whether the regulation of protein-protein interactions through AS-mediated removal/substitution of the binding site is limited to specific examples or conversely represents a widespread phenomenon. To this end we evaluated in a systematic way the extent of the overlap between alternatively spliced regions and binding residues in human proteins. In principle the overlap could be significantly higher (i.e. AS preferentially targets protein-protein interfaces), lower (interfaces are “protected” from AS), or not significantly different from expectation.

Similar studies have been conducted in the past, but we argue that a systematic statistical analysis is still lacking. A work published in 2004 [17] showed that protein-protein interface residues, extracted from complexes of known structure, are not preferentially spliced nor protected from AS. However, this work was based on too small a number of cases to draw general conclusions (16 proteins involved in heterodimeric interactions and 5 involved in homodimeric interactions). In 2006 Yura and coworkers analyzed 242 alternatively spliced regions covering less than 100 amino acids and found that in 57% of cases they partially or totally co-localize with functional sites of human proteins - defined as protein-protein, protein-ligand and protein-DNA/RNA binding sites [18]. However, the authors did not try to assess the statistical significance of this result.

Here we perform a systematic study of the relationship between AS and protein-protein interfaces in the human proteome. First we created two data sets, composed of proteins whose genes undergo alternative splicing and that are involved in heterodimeric (431 binding sites) or homodimeric (763 binding sites) interactions, as defined from complexes of known structure. We then demonstrated that, for both classes of interaction, the removal of a portion of the interacting site occurs less frequently than expected under a random model. This tendency is maintained even if we filter out isoforms that are extremely different from the canonical one and that are likely to produce drastic structural changes. We also found that hot spot residues, i.e. the ones that contribute most to the binding energy [19,20], do not behave differently from the rest of the interface. Finally, we found that the relationship between AS and interface residues does not depend on the functional class of the protein.

## Results and discussion

### Data sets creation

We first created a dataset of human protein-protein interfaces, starting from complexes of known structure in the Protein Data Bank (PDB) [21]. Using a simple distance criterion to identify interface residues, we obtained a set of 2763 heterodimeric and 5846 homodimeric interfaces in structures that have a good coverage of the corresponding protein sequences. We will use the term “semi-interface” to refer to a group of interface residues belonging to one of the interacting chains. We then selected all the proteins that could be mapped to a transcript with at least one alternative splicing isoform, thus retaining 2757 heterodimeric semi-interfaces and 4356 homodimeric interfaces. These data sets are redundant because of two main reasons: i) the same interaction can be represented multiple times, not only in different PDB structures but also within the same complex, e.g. in the case of a homomultimer having more than two chains; ii) similar (paralogous) proteins can interact with the same partner by using homologous residues [22]. In the latter case, even though the pattern of alternative splicing rapidly diverges after gene duplication [23], the way in which alternative splicing acts on related interfaces could remain similar, thus introducing some bias in the statistical analysis. To remove redundancy we selected a single representative for each group of interfaces (or semi-interfaces in the case of heterodimers) that are similar and belong to similar or identical proteins. We thus obtained a non-redundant data set composed of 431 heterodimeric semi-interfaces and 763 homodimeric interfaces. The heterodimeric semi-interfaces we found are mapped to 305 distinct UniProt [24] entries, while the homodimeric interfaces to 520 Uniprot entries. A total of 754 and 1304 distinct alternative isoforms were found for the former and the latter class of proteins, respectively. The Uniprot entries were used as reference isoforms, on which the AS changes observed in the alternative isoforms were mapped. Additional file 1 contains the list of proteins involved in heterodimeric interactions, together with the alternative splicing isoforms and the number of interface residues they remove, while the Additional file 2 contains the information about the homodimeric interactions.

### Protein-protein interfaces are protected from alternative splicing

**Figure 1:**
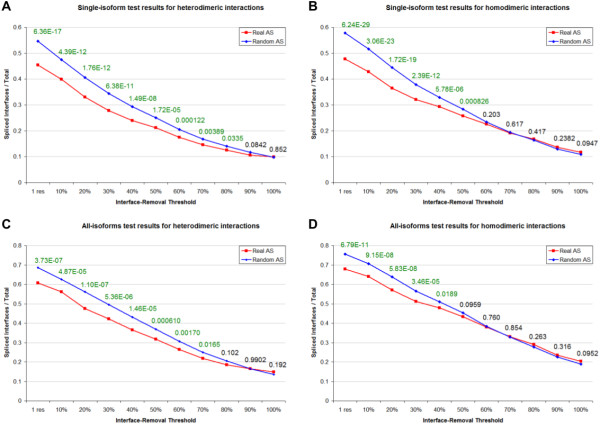
**Alternative splicing-mediated partial removal of protein interfaces is less frequent than expected.** The results obtained for the single-isoform (**A** for heterodimeric interactions and **B** for homodimeric interactions) and for the all-isoforms tests (**C** for heterodimeric interactions and **D** for homodimeric interactions) are summarized here. For each value of the interface-removal threshold, the frequency at which an interface is removed by real splicing (red line) and by randomized splicing (blue line) is reported, along with the p-value of the test (green is significant).

We sought to determine whether the removal of protein-protein interfaces via alternative splicing occurs at higher or lower frequencies compared to what happens under a random model of splicing. To do this, we followed two alternative approaches, evaluating the splicing of the binding site in each isoform separately (single-isoform test) or in each group of isoforms from the same gene (all-isoforms test). Due to the different nature of the interaction, these tests were performed on heterodimeric semi-interfaces and homodimeric interfaces separately. Indeed, in the first case the alternative splicing of a gene can act only on a single semi-interface, while in the latter there are two semi-interfaces, which differ by varying degrees, that can be spliced away. In the single-isoform test we asked whether the presence or absence of the interface in a specific isoform can be attributed to chance, taking into account the splicing pattern. If this hypothesis is rejected, one can argue that the inclusion or removal of the interacting region might be the result of some evolutionary pressure. To perform this test, we considered every heterodimeric semi-interface/isoform pair and assessed whether the isoform contains the interface region or not. An heterodimeric semi-interface is regarded as removed if the isoform does not contain at least a certain fraction of its residues. Since it is not easy to determine what is the minimum percentage of interface residues that must be spliced away in order to abolish the interaction, we repeated the test using different values for this interface-removal threshold. In order to derive a random background against which to evaluate the significance of the observed removal rate, we created 1000 decoys for each isoform (used as controls for the test), using a procedure that takes into account the specific splicing pattern of the isoform (see Methods for details). We then performed a Cochran-Mantel-Haenszel (CMH) chi-square test [25] to determine whether the frequency at which alternative splicing removes the interfaces is significantly different from that obtained using the decoys. The results of this test performed on heterodimeric semi-interfaces, using different interface-removal thresholds, are summarized in Figure 1A. The percentage of interface/isoform pairs in which the interface is removed via alternative splicing is quite high (e.g. in about 28% of the cases 30% or more of the interface is removed), but it is still significantly lower than what is observed under a random model of splicing. This difference decreases as the threshold values increases, to become almost zero when the threshold value is equal to 100%. The differences observed using threshold values <= 80% are significant (p-value < 0.05), and the significance is higher for lower thresholds. These results suggest that alternative splicing tends to avoid partial removals of heterodimeric interaction sites, while being quite indifferent to large excisions - possibly because they occur along with major rearrangements of the protein, in which cases there might be no pressure in maintaining the interaction. The single-isoform test for homodimeric interfaces was performed in a similar way, the only difference being that in this case an interface is considered removed if at least one of the two semi-interfaces lacks a certain percentage of its residues or more. As shown in Figure 1B, also in this case partial removals of the interface are significantly prevented, though the significance is lost at a lower threshold compared to heterodimers. Furthermore, really large interface excisions occur more frequently, but not in a significant way.

Using the single-isoform test, we can analyse the selective pressure towards interface retention for each individual isoform. However we underestimate all the cases in which the abolishment of the interaction occurs only in one or few isoforms among all those produced by the same gene, while the others are still able to interact, or at least they maintain the interface residues; for example, if a gene has 5 isoforms, but only one lacks the binding site, the signal coming from that lone isoform is masked by the other four. Moreover, different isoforms from the same gene can depend on the same splice junctions, hence the interacting proteins with the largest number of splicing isoforms contribute the most to the final result, not only because they are associated to a higher number of contingency tables in the CMH test (see Methods for details), but also because the splicing patterns of their isoforms are somewhat dependent. For these reasons, we performed another test, called all-isoforms test, whose purpose was to determine how likely is for an interface to be selectively removed in at least one isoform. In this case, for each interacting protein we created 1000 random controls, each consisting of a group of decoy isoforms all derived from the real isoforms of its gene. A CMH test was employed to determine whether the frequency at which a binding site is removed in at least one isoform is significantly different from that obtained under this random model of AS. The test showed a significant protection form partial removals both for heterodimeric (Figure 1C) and homodimeric (Figure 1D) interfaces, even if homodimeric interfaces appear to tolerate larger removals compared to heterodimeric ones (again, the frequency at which they undergo large removals is higher than that of the background, but not in a statistically significant way). Ultimately, the possibility of regulating protein-protein interactions through the production of an isoform which specifically lacks part of the interface region is realized less than expected; the excision of an interface is not so uncommon *per se*, since, for example, about 42% of the heterodimeric semi-interfaces undergo a removal of 30% or more of their residues in at least one isoform, but there is a general tendency towards the protection of the binding sites from AS.

### Interface protection is maintained in similar isoforms

The set of isoforms we used includes a number of transcripts that are markedly different from the canonical isoform, i.e. the isoform that corresponds to the PDB chain. These conspicuous changes are likely to result in the disruption of the 3D structure or in the production of a new structural conformation [26]. Therefore, even if the interface region is not removed, it is reasonable to assume that these isoforms would hardly maintain a conformation that allows the interaction. Hence, we repeated the previously described statistical analyses including only isoforms that maintain most of the sequence of the canonical protein. We used a variable threshold for this coverage value (from 0 to 100% in increments of 10%). Both for heterodimeric and homodimeric interfaces, the results of the two tests showed again a significant protection from partial removals, and this was true irrespective of the threshold values (these results are shown in the Additional file 3). The tendency for alternative splicing to avoid interfaces is thus confirmed in those isoforms that are more likely to be involved in a fine regulation of the interaction.

### Hot spots are not protected more than other interface residues

Since some interface residues are more important than others for the interaction to be established, we sought to determine the behaviour of alternative splicing towards these protein-protein interaction hot spots. Again, we employed the single-isoform and the all-isoforms tests, this time considering only those heterodimeric interface residues that were predicted to be hot spots (15 interfaces do not contain any hot spot, and were not included in the analysis). The results are very similar to those obtained using all the interface residues (Additional file 4). However, it was necessary to establish whether hot spots are more protected than other interface residues or not. To do so, we tested the null hypothesis that, given a heterodimeric semi-interface/isoform pair, the splicing of an interface residue is independent of whether it is a hot spot or not—again, this was done by using a CMH test. Hence, although it has been shown that hot spots are often spatially arranged in clusters called “hot regions” [27], and a manual control of the distribution of the hot spots on the sequence revealed that they tend to form clusters also in the primary structure, the protection from partial removals of the interacting region is not selective towards these most important residues. Possibly this occurs because any rearrangement of the interacting site is likely to have some influence on the interaction or, more in general, on the functionality of the protein.

### Functional significance of interface protection/removal selectivity

Using the number of random controls that removes an interface, it is possible to quantify how selectively targeted is the removal or the protection of the interface residues (see Methods for details). We selected proteins in which removal/protection of the binding site is more specific, and determined whether these two classes of proteins differ by properties that can be described using Gene Ontology (GO) [28] terms. To this end we evaluated the enrichment of GO terms in all the three main ontologies (“Molecular Function”, “Biological Process” and “Cellular Component”) for both classes of proteins. No significant enrichments were observed both for the heterodimeric and the homodimeric interactions, suggesting that the protection of the interface is a widespread phenomenon among proteins with different characteristics and that the selective removal of the interface is not preferentially restricted to a specific group of proteins sharing the same function or localization.

**Figure 2:**
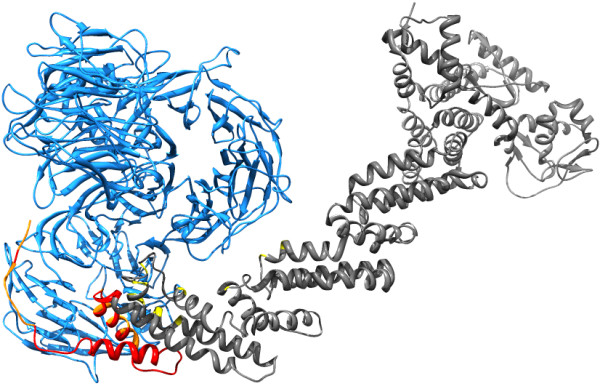
**CUL4A interacting with DDB1 using its N-terminal region, which is partially removed by alternative splicing.** The protein on the right is CUL4A. The region that is missing in the alternative isoforms is colored in red. The residues of CUL4A that interact with DDB1 and that are located in the alternatively spliced region are colored in orange, while the others in yellow. The other proteins present in the crystal are not included.

**Figure 3:**
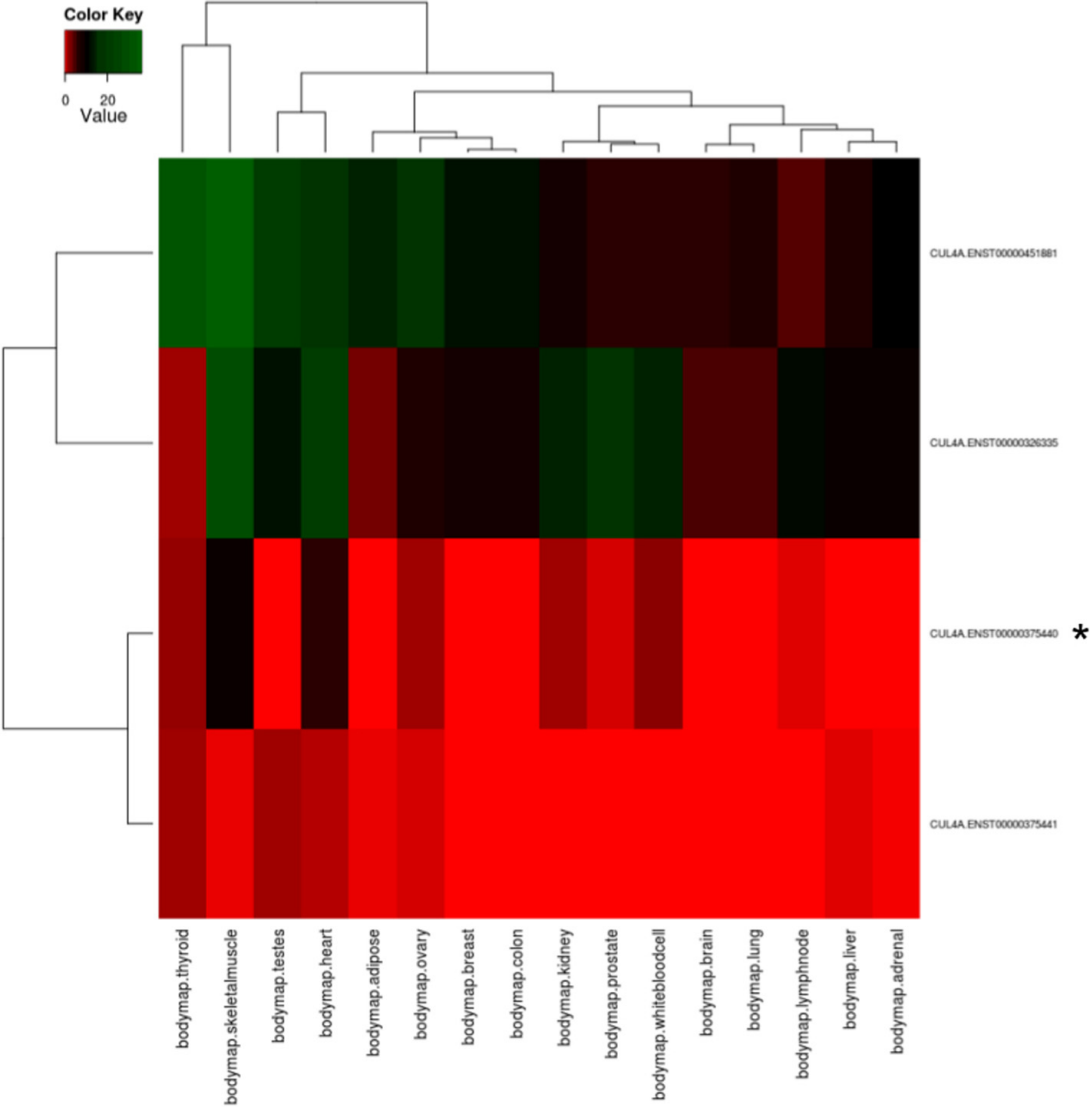
**CUL4A isoforms show different expression patterns.** The heatmap reports the expression levels (FPKM) of the transcripts using a color scale and grouping the expression patterns by similarity. The row referred to the canonical transcript is marked with an asterisk.

Though the results of this work suggest that interfaces are protected from AS, there are some cases in which the alternative splicing of the pre-mRNA is focused on the interacting region, as reported in Additional file 5 (heterodimers) and 6 (homodimers). We searched literature to investigate some of these cases, but we did not find any example in which the involved isoform has been characterized as non-interacting. However, we discovered a potential example of this regulation, involving the interaction between Cullin 4A (CUL4A) and DNA damage-binding protein 1 (DDB1). Together with ROC1, which interacts with CUL4A, these proteins form a complex (PDB: 2hye [29]), which acts as an E3 ubiquitin ligase that, among other things, participates in the degradation of DNA damage-response proteins [30-34]. The N-terminal region of CUL4A is bound to a beta-propeller domain of DDB1. The CUL4A pre-mRNA undergoes alternative splicing: the protein observed in the crystal can be mapped to the Ensembl [35] ENST00000375440 (the “canonical” transcript), while three other full-length coding transcripts exist, which share the same coding sequence. The only difference between the coding sequence of the alternative transcripts and that of the canonical one is that the former lacks the first 100 residues of the latter. Figure 2 shows the interaction between CUL4A and DDB1 and highlights the region of CUL4A that is missing in the alternative isoforms. To our knowledge, the functionality of the alternative isoforms has not been well characterized. However it is quite likely that, since they lack 19 of the 30 semi-interface residues, they are not capable of binding DDB1. Indeed it has been reported that the deletion of the first 97 amino acids of the canonical protein completely abolishes DDB1 binding [36]. Despite the general trend towards protection of interfaces from AS, in this case we observe a selective partial removal of the interface, and this is likely to result in the production of protein isoforms that cannot bind to a specific partner. We also investigated the tissue-specific expression patterns of the transcripts encoding CUL4A. As shown in Figure 3, the canonical isoform is expressed only in some tissues, while the expression of the alternative transcripts taken together covers all the tissues and is higher than that of the canonical transcript in the tissues where it is present. Hence, in addition to other regulatory processes that might influence the interaction, alternative splicing seems to provide a mechanism through which the association of CUL4A with DDB1 is only possible in a specific subset of human tissues.

## Conclusions

The present study demonstrates that the removal of protein-protein interfaces by AS is generally avoided in the human proteome, and this holds true for both heterodimeric and homodimeric interactions. These results are not in disagreement with previous works demonstrating that protein-protein interaction domains and protein interaction-associated linear motifs are significantly found in alternatively spliced regions [8,9,11]. Indeed, in the analysed dataset, only ~37% of the heterodimeric semi-interfaces and only ~45% of the homodimeric interfaces we analysed are fully included in a PFAM [37] domain. Moreover, putative SliMs (i.e. short linear motifs) interacting with domains are present in very low amounts in our data set. We used the iELM web server [38,39] to analyse our lists of heterodimeric and homodimeric interacting protein pairs and predict SliM-mediated interactions, and we found that only 6 out of 431 heterodimeric semi-interfaces and 19 out of 763 homodimeric interfaces contain at least one residue belonging to a putative domain-binding SliM. We additionally verified whether the protein interaction interfaces in our datasets overlap with protein disordered regions, since it is known that AS events are preferentially located in such locations [40,41]: these regions are enriched in functional motifs [42,43] and are more tolerant to the structural changes introduced by AS. Since they are derived from the three-dimensional structures of crystallized proteins, our binding sites are almost totally localized outside disordered regions: only 16 heterodimeric semi-interfaces and 26 homodimeric interfaces have 30% or more of their residues in a putative disordered conformation (to obtain the information about the disordered regions we extracted the consensus predictions stored in the Database of Disordered Protein Predictions [44]). This is due to the fact that these sites are derived from the three-dimensional structures of crystallized proteins; furthermore, we filtered out those crystallized dimers in which the two interacting chains cover less than 30% of the complete protein sequence, thereby eliminating the interactions mediated by small peptides, which are often embedded into disordered regions. Given all these results, it is clear that the feasibility of an AS-mediated removal of the binding site depends on the structural characteristics of the interaction, since, for instance, it is easier to modulate the inclusion of SliMs, which are often localized in disordered regions, than it is to remove or substitute binding sites that reside in structurally defined regions. In any case, these considerations do not concern those cases in which the regulation of the interaction does not consist in a direct removal/substitution of the region involved in the interaction (which can be a domain, a motif or an interface), e.g. when a sequence is inserted within a binding domain.

Our findings are confirmed even when we consider only those alternative transcripts whose coding sequence is only slightly different from that of the canonical isoforms. Moreover, the protection from alternative splicing is not limited to the interface residues that significantly contribute to the binding free energy, thus emphasizing the importance of maintaining the global architecture of the binding site. This requirement seems to be common to proteins with different properties: the proteins in which alternative splicing selectively avoids the binding site were not found to be prevalently associated to any specific function, biological process or localization. This was also observed for those proteins in which the binding site is selectively removed in one or more isoforms. We studied one of these cases, represented by the partial removal of the region through which Cullin 4A interacts with DDB1, and found that the isoforms lacking the binding site show an expression pattern that greatly differs from that of the interacting protein. Recent works show how the tissue-specific AS of protein-protein binding sites, especially when disordered, has an important role in rewiring protein-protein interaction networks in a tissue-specific fashion [11,45]. As a further extension of our study, we are currently analysing splicing isoform-specific expression patterns in combination with our dataset of binding sites, which reside mostly outside disordered regions, in order to understand how isoforms containing a different portion of a structured binding site (or lack thereof) are used in different tissues and/or conditions.

## Methods

### Identification of human protein-protein interfaces

To obtain a set of human protein-protein interfaces, we searched for all the x-ray PDB structures containing two or more human protein chains, thus obtaining a set of 6637 PDB structures. We retained only those chains that could be unambiguously assigned to UniProt entries according to SIFTS [46] (about 93% of the total). The SIFTS resource was also used to obtain a residue-level mapping between PDB and UniProt sequences. We employed the HotPOINT [47] algorithm to fetch interface residues; some of these residues were classified as hot spots by the program, however, since the program has been trained on a set composed of only heterodimers, we rely only on the hot spots predicted for this kind of interaction. The distance threshold for the identification of the interacting residues was set to the default value (two residues belonging to different chains are assumed to interact if there is at least one atom from one residue that is at a distance from any atom of the second residue at most equal to the sum of Van der Waals radii of the two atoms + 0.5Å). To avoid crystallographic artifacts, the search for protein-protein interaction interfaces was conducted only on those pairs of protein chains of the Asymmetric Unit that were present with the same relative orientation in at least one of the Biological Units assigned by the authors of the structure. Among these pairs, 13136 interact with at least one pair of residues mapped on the UniProt sequence. We took only the interfaces in which both chains interact with at least five residues, thus obtaining a set of 11555 dimers. Moreover, we filtered out all the dimers in which the sequence of one or both chains does not cover at least 30% of the corresponding UniProt sequence.

### Alternative splicing data retrieval

The genes coding for the interacting proteins were found using Biomart [48] to mine Ensembl cross-references. Out of 1167 distinct proteins, 1113 were unambiguously assigned to an ENSG (i.e. an Ensembl gene). Each of them was aligned with all the transcripts associated to the corresponding gene using the Needleman-Wunsch algorithm [49]. For 1057 proteins it was possible to find one or more ENSTs (i.e. the Ensembl transcripts) with identical coding sequence. The remaining proteins were associated to the most similar transcripts, provided that there were up to five non-contiguous gaps or substitutions (28 proteins did not satisfy this condition). For each of the 1085 proteins that were assigned to a transcripts, we took all the other full-length protein-coding ENSTs belonging to the same ENSG as alternative splicing isoforms; those transcript that are subject to nonsense-mediated decay according to Ensembl were not took into consideration. This way we obtained a set of 804 proteins whose genes encode for one or more alternative splicing isoforms distinct from the reference protein sequence.

### Redundancy reduction

To perform a clustering of the interfaces and to select one representative for each interface, we first grouped together similar proteins using the BLASTClust software [50], which is based on BLAST [51]; two proteins were clustered together if they had >= 30% sequence identity over an area covering at least 90% of each sequence. The 804 proteins whose genes undergo alternative splicing resulted divided into 723 similarity groups. For each group of similar sequences, a multiple sequence alignment was performed using T-coffee [52]. These alignments allowed us to compare interfaces from proteins belonging to the same similarity group, since each interface residue could be mapped to the multiple alignment and identified with its alignment column number. All the heterodimeric semi-interfaces of proteins within the same cluster were clustered using a hierarchical complete linkage algorithm with distance measure 1 – O, where O is an overlap value defined as the number of identical residue identifiers between two semi-interfaces divided by the number of residues of the smallest semi-interface. To obtain the clusters, the clustering tree was cut at a height equal to 0.5: this way we grouped together semi-interfaces with O >= 0.5. This clustering procedure was inspired by a work from Bordner and Gorin [53]. A similar method was used to cluster homodimeric interfaces. In this case, O is defined as the number of identical residue identifiers between two interfaces divided the number of residues of the smallest interface. The numerator can have two different values, because one of the two semi-interfaces from one dimer can be compared with both the semi-interfaces from the other dimer; for each pairs of interfaces we have chosen the combination that maximizes the numerator.

For each cluster we elected a representative (semi-)interface by applying these criteria:

– Choose the interface associated to the chain that most covers the UniProt sequence (in the case of homodimers we use the product of the coverage of the two interacting chains);

– If multiple interfaces meet the above criterion, choose among them the interface found in the PDB entry with the best resolution;

– If multiple interfaces meet the above criterion, choose among them the interface that has the largest number of residues;

– If multiple interfaces remain, choose among them randomly.

### Statistical tests

In all the employed variants of the statistical analysis of AS-mediated interface removal, each isoform was described as a two-bit string, in which to each residue of the interacting protein we assigned value 0 if it is found in that isoform, or 1 if it is missing. We define as “missing stretch” every stretch of contiguous 1 characters, which is a contiguous protein region removed by splicing events. A region between two missing stretches will be called “non-missing stretch”, while the region that spans from the first missing stretch to the last one (or that coincides with the only missing stretch) will be referred to as “variable region”. The alignment between the transcript coding for the interacting protein and each isoform was done using the genomic coordinates of the coding sequences provided by Ensembl.

**Figure 4:**
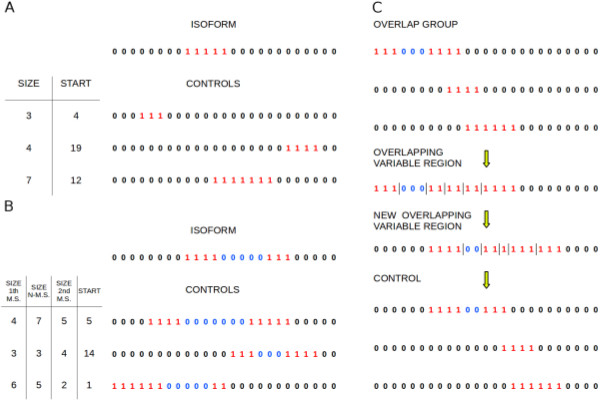
**Toy examples of control creation for a single-isoform test (A and B) and for an all-isoforms test (C). (A)** The isoform of an interacting protein for which controls are created is represented as a two-bit string, in which 0 describes a conserved position (i.e. not removed by a splicing event) and 1 a non-conserved position. A stretch of 1s is called “missing stretch” (M.S.) and is coloured in red. In this example the isoform has one missing stretch. Controls are created by randomly changing the number of residues (size) of the missing stretch according to a Poisson distribution with mean equal to the real size of that stretch and randomly selecting the starting point of the stretch. **(B)** The isoform has two missing stretches and the region between them is called “non-missing stretch” and is coloured in blue. The region that encompasses all the missing and non-missing stretches is called variable region. Again, controls are created changing the size of the missing stretches, but also of the non-missing stretches (N-M.S.) and by randomly selecting the starting point of the variable region. **(C)** A set of isoforms forming an overlap group, i.e. a group in which, for each isoform, the missing stretches partially or totally overlap with those of one or more isoforms from the same group. All the variable regions in the overlap group are fused into an “overlapping variable region” divided into segments by the junctions between missing stretches and non-missing stretches or regions outside variable regions, represented as pipes in the figure. Each segment's size is randomly changed according to a Poisson distribution with mean equal to the segment's size, then the starting point of the newly formed overlapping variable region is randomly selected.

In the single-isoform test, each control represents a decoy isoform. These controls were created by giving each missing or non-missing stretch a new size (number of residues) obtained by picking a random value from a Poisson distribution with parameter λ equal to the real size of that stretch and choosing randomly the starting position of the newly formed variable region (the order of the stretches in the variable region remains the same). This way of creating decoys by pulsing missing and non-missing stretches allows us to generate a large set of different controls for each isoform without radically changing the number, the dimension and the relative position of the alternatively spliced regions, and as a consequence the manner in which they can act on the interface. Figure 4 shows an example of control creation from an isoform with one (a) or two (b) missing stretches. After creating 1000 controls for each isoform, we compared the frequency at which they lack the binding site with the real frequency of AS-mediated interface removal using a CMH test. In this test, for each (semi-)interface/isoform pair, a 2×2 contingency table is compiled, which describes the variables “isoform” (real or decoy) and “interface removal” (interface is removed or not, at a given interface-removal threshold). Table 1 is an example of contingency table. For each table, the absolute value of the difference between the observed and the expected value of one cell, and the deviation between observed and expected values are computed. These values, obtained from all the tables, are then combined to determine whether the two variables are dependent or not. We employed this approach instead of using a chi-square test with a single contingency table because in this way controls are compared only with the isoform from which they were derived.

**Table 1:** Example of a 2×2 contingency table computed for an interface/isoform pair in the single-isoform test

	**Interface is removed**	**Interface is not removed**
Real isoform	1	0
Decoy isoform	225	775

In this example the (semi-)interface is removed in the real isoform and in 225 out of 1000 decoy isoforms.

In the all-isoforms test, each control consists in a group of decoy isoforms of the interacting protein, each one made from one of the real alternative transcripts. In this case the overlap between missing stretches of different isoforms has to be maintained: this way the extension of the regions of the interacting protein that are alternatively spliced does not vary much, the only source of variation being the pulsation of the missing stretch. To create a control for a given protein, we first divided the set of isoforms of that protein in subgroups called “overlap groups”, such that every isoform in an overlap group had at least one missing stretch totally or partially overlapping with the missing stretches of one or more isoforms belonging to the same group, but not to other groups; each isoform with no overlapping partners was assigned to its own single-member overlap group. For each overlap group, an “overlapping variable region”, ranging from the minimum starting position to the maximum ending position among all the variable regions of the isoforms from that group, was identified. All the junctions between a missing and a non missing stretch or between a missing stretch and a region outside the overlap group were used to split the corresponding overlapping variable region into segments: each segment is a partial or entire missing or non-missing stretch that is shared among a specific sub-group of isoforms belonging to the overlap group. For each overlap group, the starting position and the size of every segment was changed by sampling from a Poisson distribution as previously described for the single-isoform test. This process is illustrated in Figure 4C. All the newly formed overlapping variable regions were used to create the control decoy isoforms, assigning each varied segment to the isoforms from which it derives, provided that:

– no pair of overlapping variable regions shows an overlap between segments derived from a missing stretch;

– since the previous condition favors the shortening of the missing stretches (shorter segments are less likely to overlap), if there are multiple overlap groups, the total number of residues in the varied missing exons must not be less than 90% of the total size of the real missing exons.

1000 controls were created for each interface. Again, we performed a CMH test, compiling for each (semi-)interface a contingency table with variables “alternative splicing” (real or control) and “interface removal” (more than a certain fraction of the isoform is removed in at least one isoform or not).

To test whether hot spots are more removed or protected than other interface residues, a CMH test was performed, assigning to each (semi-)interface/isoform pair a contingency table in which the number of hot spot and non-hot spot residues that are removed or not were reported.

All the statistical tests were performed using R version 2.14.2 [54].

### Assessment of the GO terms enrichment

For each interface-removal threshold value, we assigned each spliced interface/isoform pair a score equal to the fraction of the single-isoform test controls that remove the interface, representing how much that interface is exposed to splicing in that particular isoform. We then selected all the proteins associated to a score lower than 0.5 (i.e. those in which the interface is selectively removed with respect to a background of splicing in at least one isoform). The interfaces that are not spliced in any isoform were assigned a score equal to the fraction of the all-isoforms test controls that remove the interface, and those having a score higher than 0.5 (i.e. those proteins whose alternative splicing selectively avoids the binding site) were selected. We employed Gorilla [55,56] to evaluate the GO terms enrichment for both lists of proteins (those having selectively spliced or selectively protected interfaces) against the proteins data set used in the test (heterodimeric or homodimeric). The evaluation of the GO terms enrichment was repeated for each value of the interface-removal threshold. Only the GO terms for which a False Discovery Rate q-value < 0.05 was obtained were considered as significantly enriched. Different cutoff values other than 0.5 were also tested, obtaining similar results.

### Evaluation of the expression of alternative transcripts

The tissue-specific expression of the CUL4A transcript variants was retrieved from DBATE (Bianchi et al., submitted; URL: http://bioinformatica.uniroma2.it/DBATE/), an online database of alternative transcripts expression that was developed in our laboratory. This database was created processing thirteen RNA-seq panels from human tissues gathered from the Gene Expression Omnibus (GEO) service [57] using the Tuxedo suite, which employs Bowtie [58], Cufflinks [59] and TopHat [60], to map the sequence reads to the human genome (hg19 assembly) and to evaluate the normalized expression of the Ensembl splicing variants reported in Fragments Per Kilobase of transcript per Million mapped reads (FPKM).

### Description of additional data files

The following additional data files are available with the online version of this paper. Additional files 1 and 2 are tables listing, respectively, all the heterodimeric semi-interfaces and the homodimeric interfaces we analyzed, along with the alternative isoforms and the number of interface residues they lack. The Additional file 3 is a set of tables summarizing the results for single-isoform and all-isoforms tests performed on both heterodimeric and homodimeric entire binding sites. The Additional file 4 is a set of tables summarizing the results of single-isoform and all-isoforms tests performed on the hot spot residues of heterodimeric binding sites. Additional files 5 and 6 are tables listing all the transcripts that selectively lack heterodimeric and homodimeric binding sites, respectively.

## Abbreviations

AS: Alternative splicing; CMH: Cochran-Mantel-Haenszel; CUL4A: Cullin 4A; DDB1: Dna damage-binding protein 1; GO: Gene ontology; PDB: Protein data bank; SIFTS: Structure integration with function taxonomy and sequence; SliM: Short linear motif.

## Competing interests

The authors declare that they have no competing interest.

## Authors’ contributions

AC did most of the analysis and wrote the paper. VB contributed to the data set creation and did the analysis of the expression levels of CUL4A isoforms. PFG and GST set up the statistical test. GA, MHC and FF conceived and coordinated the study. All authors read and approved the final manuscript.

## Supplementary Material

Additional file 1 **AS of heterodimeric interfaces.** This table lists, for each PDB chain involved in a heterodimeric interaction, the chain name of its partner, the corresponding UniProt Accession Number and ENSTs, the ENSTs of the alternative isoforms with identical coding sequence, along with the fraction of interface and hot spot residues missing in those isoforms. Click here for file

Additional file 2 **AS of homodimeric interfaces.** This table lists, for each pair of PDB chains involved in a homodimeric interaction, the corresponding UniProt Accession Number and ENSTs, the ENSTs of the alternative isoforms with identical coding sequence, along with the fraction of interface residues of both chains missing in those isoforms. Click here for file

Additional file 3 **Single-isoform and all-isoforms tests results for heterodimeric and homodimeric interfaces.** This files include all the tables summarizing the results of single-isoform and all-isoforms tests performed on heterodimeric and homodimeric interfaces. The results of the tests performed only with isoforms whose sequences cover at least a certain fraction of the canonical ones are also reported. Threshold is the interface-removal threshold value used in the test. Spliced in real AS/Not spliced in real AS is the number of isoforms that remove or not a binding site (in the single-isoform test) or of binding sites that are removed or not in one or more isoforms (in the all-isoforms test). Spliced in random AS/Not spliced in random AS is the number of decoy isoforms that remove or not a binding site (in the single-isoform test) or of binding sites that are removed or not in one or more decoy isoforms (in the all-isoforms test). Spliced in real AS fraction is the second column/ (second column + fourth column) fraction. While Spliced in random AS fraction is the third column/ (third column + fifth column) fraction. P-value is the p-value obtained for the CMH test. Click here for file

Additional file 4 **Single-isoform and all-isoforms tests results for heterodimeric interfaces hot spots.** This file includes all the tables summarizing the results of single-isoform and all-isoforms tests performed on the hot spot residues of heterodimeric binding sites. See the legend of Additional file 3 for a description of the columns. Click here for file

Additional file 5 **Selectively spliced heterodimeric semi-interfaces.** This table lists the ENSTs of all the transcripts that selectively remove a heterodimeric semi-interface. Click here for file

Additional file 6 **Selectively spliced homodimeric interfaces.** This table lists the ENSTs of all the transcripts that selectively remove a homodimeric interface. Click here for file
